# Identification of DNA Methylation Changes That Predict Onset of Post-traumatic Stress Disorder and Depression Following Physical Trauma

**DOI:** 10.3389/fnins.2021.738347

**Published:** 2021-09-24

**Authors:** Carina A. Martin, Rany Vorn, Martin Schrieber, Chen Lai, Sijung Yun, Hyung-Suk Kim, Jessica Gill

**Affiliations:** ^1^National Institute of Nursing Research, National Institutes of Health, Bethesda, MD, United States; ^2^Division of Trauma, Critical Care and Acute Care Surgery, Oregon Health and Sciences University, Portland, OR, United States; ^3^Yotta Biomed, Bethesda, MD, United States

**Keywords:** post-traumatic stress disorder, major depressive disorder, DNA methylation, epigenetics, biomarkers

## Abstract

Post-traumatic stress disorder (PTSD) and major depressive disorder (MDD) are commonly experienced after exposure to highly stressful events, including physical trauma, yet, biological predictors remain elusive. Methylation of DNA may provide key insights, as it likely is reflective of factors that may increase the risk in trauma patients, as DNA methylation is altered by previous stressors. Here, we compared DNA methylation patterns using bisulfite sequencing in patients with a physical trauma that required more than a 24-h hospitalization (*n* = 33). We then compared DNA methylation in patients who developed and compared the following groups (1) PTSD and MDD; *n* = 12), (2) MDD (patients with MDD only; *n* = 12), and (3) control (patients who did not have PTSD or MDD; *n* = 9), determined by the PTSD Checklist (PCL-5) and Quick Inventory of Depressive Symptomatology (QIDS) at 6-months follow-up. We identified 17 genes with hypermethylated cytosine sites and 2 genes with hypomethylated sites in comparison between PTSD and control group. In comparison between MDD and control group, we identified 12 genes with hypermethylated cytosine sites and 6 genes with hypomethylated sites. Demethylation of these genes altered the CREB signaling pathway in neurons and may represent a promising therapeutic development target for PTSD and MDD. Our findings suggest that epigenetic changes in these gene regions potentially relate to the onset and symptomology of PTSD and MDD and could be used as potential biomarkers in predicting the onset of PTSD or MDD following traumatic events.

## Introduction

Post-traumatic stress disorder (PTSD) and major depressive disorder (MDD) are common psychiatric disorders that can develop in individuals who have experienced exposure to extreme stress or a traumatic event (Klengel et al., 2014; Ryan et al., 2016). PTSD is often comorbid with MDD, and many of the symptoms of these conditions overlap. The similarities in the phenotypes of these comorbid disorders make the diagnosis of PTSD and MDD difficult because the clinical diagnosis of these disorders relies on subjective measures (Weathers et al., 2013; Blacker et al., 2019). Therefore, it is not clear how the onset of PTSD occurs, making it very difficult to predict and develop methods for prevention (Qi et al., 2016). This is a critical issue, as PTSD and MDD have been linked to flashbacks, nightmares, avoidance behaviors, negative mood/thoughts, and/or alterations in arousal, which include exaggerated startle, and sleep disturbance (Klengel et al., 2014; Blacker et al., 2019). Both conditions cause major impacts on quality of life, and often, inhibit a person’s ability to participate in activities they enjoy. PTSD and depression have been associated with lower quality of life in military personnel (Gill et al., 2014) and suicidal behavior (Sareen et al., 2007).

Identifying biological alterations that are associated with PTSD and MDD are imperative to expanding our current understanding of PTSD and MDD onset, as well as an essential step in developing more personalized methods of treatment and prevention. Increasing evidence of biomarkers research is making significant impact in the diagnosis and prognosis of psychiatric disorders, including PTSD and MDD. Many techniques have been used to identify biomarkers, including genetic, epigenetic, and protein concentration (Gill et al., 2014; Guardado et al., 2016; Martin et al., 2018). We previously reported the upregulation of inflammatory cytokines genes and plasma proteins levels in military personnel with PTSD and MDD (Gill et al., 2014; Guardado et al., 2016). However, biomarkers for PTSD and MDD are not well understood due to their complexities of the disease pathogenesis. Among individuals who develop PTSD and MDD, some have been found to be more highly susceptible than others. The variability in clinical phenotype is the result of several factors: the nature, intensity, and duration of the trigger event, genetic predisposition, epigenetics, and environmental conditions (Ryan et al., 2016; Blacker et al., 2019).

Epigenetic profiles have been used widely to identify biomarkers for several diseases, including PTSD and MDD, because they are independently associated with the regulation of gene expression (Blacker et al., 2019; Morrison et al., 2019). Whole genome sequencing after bisulfite conversion can be used to identify which cytosines are methylated genome-wide. In this pilot study, to identify DNA methylation biomarkers specific to PTSD and MDD onset, we performed bisulfite sequencing on peripheral blood mononuclear cells (PBMCs) collected from PTSD with or without MDD.

## Materials and Methods

### Study Participants

Adult patients hospitalized for at least 24 h at Oregon Health and Sciences University (OHSU) for various types of physical trauma were recruited. Initial blood draws (Day 1) were taken immediately upon consent of the patient, and second blood draw (Day 2) was taken 24 h following the first blood draw. A clinical interview was used to exclude patients with previous PTSD or MDD, or major neurological disease, suicidality, or medical conditions that would compromise ability to fully provide consent. Patients were included irrelevant of primary identified language. PBMCs were isolated from whole blood samples at OHSU and transferred to the National Institute of Nursing Research (NINR) lab and stored in −80°C freezer prior to use for this study.

During their follow-up visits, which occurred 1-, 3-, and 6-months post-trauma, patients were given the PTSD Checklist (PCL-5) to test for symptoms of PTSD and the Quick Inventory of Depressive Symptomatology (QIDS) as an assessment for depressive symptoms (Rush et al., 2003). The PCL-5 is a 20-item self-report PTSD symptom with scores ranging from 0 to 80. According to National Center for PTSD, the cutoff scores for probable PTSD diagnosis is 33 (Weathers et al., 2013). In the present study, patients with PCL-5 over 40 were considered to have PTSD while QIDS over 5 were considered to have MDD (Rush et al., 2003). Based on the clinical symptoms at 6-months, 33 patients were selected and classified them into 3 different groups: (1) PTSD + MDD (patients with PTSD and MDD; *n* = 12), (2) MDD only (patients with MDD only; *n* = 12), and (3) control (patients who did not have PTSD or MDD, *n* = 9). The study was conducted according to the guidelines of the Declaration of Helsinki, and approved by the Institutional Review Board of Ethics Committee at Oregon Health and Sciences University (protocol code 00812, approved 06/2014).

### Bisulfite Sequencing

Accel-NGS^®^ Methyl-Seq DNA Library kit was used to prepare the libraries from PBMCs. Illumina’s NovaSeq-6000 sequencer was used for paired-end sequencing of read length of 151 bp. Average number of bases sequenced per sample was 262 billion bases with standard deviation of 37 billion bases. Average coverage per sample was 87.4x of whole human genome with standard deviation of 12.3x.

### Bioinformatic Analysis

The quality of sequencing was evaluated using FASTQC version 0.11.9 (Simon Andrews, 2020). BISMARK version 0.22.1 was used to perform DNA methylation calls (Krueger and Andrews, 2011). The human genome, hg38, was indexed using bismark_genome_preparation module. Alignment was performed in a paired-end mode using BISMARK. Deduplication of reads was performed using duplicate_bismark module. Bismark_methylation_extractor module was used to extract methylated sites. RnBeads version 2.8.0 was used to perform differential methylation analysis with R version 4.0 (Müller et al., 2019; R Core Team, 2019). Input files were prepared by unzipping the bismark.cov.gz files obtained by running Bismark. A sample annotation file was prepared for each differential methylation comparison. RnBeads2’s rnb.run.analysis function was used to execute the differential methylation analysis. Supplementary Figure 1 shows the flowchart of differential methylation analysis. Benjamini-Hochberg’s False Discovery Rate of 0.05 was used as the cutoff of the differentially methylated sites with statistical significance. For all the cases of PTSD or MDD vs. Control, we aggregated Day1 and Day2 to detect dysregulated DNA methylation markers averaged over the 2 days to accommodate the variability in the 24-h time period. To identify biomarker methylated genes for PTSD and MDD or MDD only with lowest false positives, we performed differential expression analysis on Day1 and Day2 for each, then, reported the commonly found genes. This work utilized the computational resources of the NIH HPC Biowulf cluster.

## Results

### Study Population and Demographics

The average age of patients was 50 years old (*SD* = 16), and the majority of patients were male. The groups did not significantly differ on age, sex, race, and injury mechanism based on the results of Chi-Square and ANOVA (Table 1).

**TABLE 1:** Characteristics of 33 patients hospitalized for trauma.

**Variable**		***Control (n* = *9)***	**PTSD or MDD Case (*n* = 24)**		***P*-value**
			**PTSD + MDD (*n* = 12)**	**MDD (*n* = 12)**	
Age (years), Mean (SD)		39.89 (19.04)	56.25 (15.47)	51.00 (13.36)	0.077
Sex, M/F, n (%)		6/3 (66.7/33.3)	8/4 (66.7/33.3)	7/5 (58.3/41.7)	0.892
Race, White, n (%)		8 (89)	11 (91.70)	12 (100)	0.526
Mechanism of injury, n (%)					
	Death	0 (0)	12 (100)	1 (8.3)	
	Physical Accident	6 (66.7)	0 (0)	11 (91.7)	
	Other	3 (33.3)	0 (0)	0 (0)	
QIDS, *M* (SD)		1.67 (1.41)	9.08 (5.64)	9.17 (3.93)	
PCL-5, *M* (SD)		15.22 (11.13)	47.66 (16.16)	7.16 (6.53)	

### Differential Methylation

#### All Cases of Post-Traumatic Stress Disorder + Major Depressive Disorder and Major Depressive Disorder Only vs. Control

To identify methylated genes relevant to all cases of PTSD + MDD and MDD only, we first performed aggregated analysis vs. the control group. To perform this analysis, we included all samples from the Day1 and Day2 together and compared them to the control. This analysis found 1,694 sites that were differentially methylated, including 294 genes with hypermethylated cytosine sites and 160 genes with hypomethylated cytosine sites (Supplementary Table 1 and Supplementary MS Excel File1). Genes included in this analysis were methionine sulfoxide reductase A (MSRA, adjusted *P*-value = 0.0238), transmembrane protein 132D (TMEM123D, adjusted *P*-value = 0.0048), and disco interacting protein 2 homolog C (DIP2C, adjusted *P*-value = 0.0023). Ubiquitin conjugating enzyme E2 L3 (UBE2L3, adjusted *P*-value = 0.0160), which we also found to be hypomethylated at the promotor region (adjusted *P*-value = 0.0164).

#### Post-traumatic Stress Disorder and Major Depressive Disorder vs. Control

To identify genes differentially methylated in the PTSD + MDD group, which is a subset of all cases (PTSD + MDD and MDD only), we compared PTSD + MDD vs. control. Common genes found in Day1 and Day2 batches were 17 genes with hypermethylated cytosine sites and 2 genes with hypomethylated sites (Table 2). Detailed information on methylated cytosine sites with fold change and adjusted *P*-values were listed (Supplementary MS Excel File1).

**TABLE 2:** Gene list of hyper- and hypo-methylated cytosine sites for PTSD + MDD vs. control.

**Genes with hypermethylated cytosine sites**	**Genes with hypomethylated cytosine sites**
CBS, ERICH1, GUSBP3, HCN2, HERC2P11, ICOSLG, LINC01342, LOC100507412, LOC100996643, MRTFA, NPIPA1, PARD3, PDE4DIP, POTEE, RHD, SPTBN1, UBE2L3	FAM225B, LOC100507412

#### Major Depressive Disorder vs. Control

In comparison between MDD and control group, we identified 12 genes with hypermethylated cytosine sites and 6 genes with hypomethylated sites common in Day1 and Day2 batches (Table 3). Detailed information on methylated cytosine sites with fold change and adjusted *P*-values were listed (Supplementary MS Excel File1).

**TABLE 3:** Gene list of hyper- and hypo-methylated cytosine sites for MDD vs. control.

**Genes with hypermethylated cytosine sites**	**Genes with hypomethylated cytosine sites**
CYP4F11, DLGAP2, DNAJC3, GTF2IRD2B, LOC102724159, NBPF4, PTPRN2, RGPD1, RHD, ROBO2, TBC1D22A, UBE2L3	ANO2, FAM182B, LINC02492, MED12L, RASA4B, RGS11

#### Post-traumatic Stress Disorder-Unique and Major Depressive Disorder-Unique Genes With Demethylated Cytosines

**FIGURE 1:**
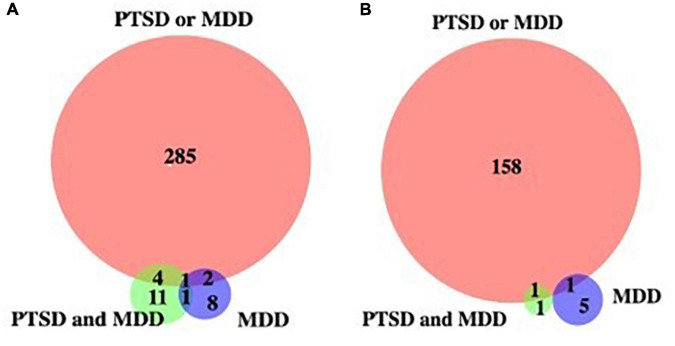
Venn diagram of genes with de-methylated cytosines. **(A)** Genes with hypermethylated cytosine sites, **(B)** genes with hypomethylated cytosine sites against control.

To identify potential biomarkers that correlates with PTSD together or with MDD without PTSD, we plotted Venn diagrams on genes with hyper- and hypo-methylated cytosine sites (Figure 1). Table 4 shows list of PTSD-unique and MDD-unique gene list with hyper- and hypo-methylated cytosine sites. The conversion rate averaged over all the samples is 0.9977 with standard deviation of 0.0011 as calculated by BCR calculator (Zhou et al., 2020). The number of CpG sites was 28.3 million as calculated by Luo et al. (2014), and the number of CpG sites in the promoter region is 3,937,537 sites.

**TABLE 4:** PTSD + MDD-unique and MDD-unique genes with de-methylated cytosines.

**Hypermethylated against control**		**Hypomethylated against control**	
**PTSD + MDD-unique**	**MDD-unique**	**PTSD + MDD-unique**	**MDD-unique**
ERICH1, GUSBP3, HCN2, HERC2P11, ICOSLG, LINC01342, LOC100507412, NPIPA1, PARD3, POTEE, SPTBN1	CYP4F11, DLGAP2, DNAJC3, GTF2IRD2B, LOC102724159, NBPF4, RGPD1, ROBO2	FAM225B	ANO2, FAM182B, LINC02492, RASA4B, RGS11

## Discussion

In the present study, we profiled genome-wide DNA methylation to identify potential candidate biomarkers for the development of PTSD and MDD onset in patients who sustained physical injuries. We report that those patients who develop either PTSD or MDD have including 294 genes with hypermethylated cytosine sites and 160 genes with hypomethylated cytosine sites, implicating the function of CREB signaling pathway in neurons which may represent a promising target for therapeutic development and intervention. We identified 12 unique genes de-methylated for PTSD and MDD vs. control group to all subjects with PTSD or MDD. These genes associated network functions of carbohydrate metabolism, lipid metabolism, and cellular function and maintenance. Specifically, relevant to MDD vs. control, we identified 13 unique genes de-methylated associated with cardiovascular disease, cellular death and survival, cellular assembly and organization network.

We also report that Ras and Rab interactor 3 (RIN3), receptor-type tyrosine-protein phosphatase 2 (PTPRN2), and long intergenic non-protein coding RNA 319 (LINC00319) genes from our prior analyses with MeDIP-seq on PTSD patients were also present as significant genes in this analysis (Martin et al., 2018). The association of these genes and neurodegenerative disorders have been reported previously (Boden et al., 2017; Shen et al., 2020). In particular, RIN3 is a guanidine nucleotide exchange factor have been significantly associated with Alzheimer’s disease (AD) pathology via endosomal dysfunction (Shen et al., 2020). PTPRN2 gene may underlie PTSD or MDD pathogenesis which may regulated neurotransmitters norepinephrine, dopamine, and serotonin (Curtis et al., 2011). The association between these genes and psychiatric conditions should be studied further, and our findings indicate that these genes could be related to PTSD as well when differentially methylated.

Among the genes that we found to be top candidates for PTSD and MDD onset the gene of most interest was UBE2L3. Its gene site was found to be differentially methylated across two of our analyses (Table 3). We also found the promoter region of this gene to be significantly hypomethylated (adjusted *P*-value = 0.016). This gene is included in a family of genes that are responsible for the ubiquitination of proteins, which is essential for targeting proteins that may be abnormal or short-lived and should be degraded. This process is especially important in that it prevents the build-up of proteins in the brain, which helps to maintain a healthy brain. UBE2L3 has been found to be associated with schizophrenia (SZ) in several studies, and one study in particular showed that this gene was found to be significantly related to phenotypic pairs of autoimmune and psychiatric conditions across multiple loci (Tylee et al., 2018). Dysregulation of UBE2L3 gene expression from the whole blood of the first episode of psychosis patients compared with healthy control populations was reported recently (Leirer et al., 2019). Dysregulation of UBE2L3 expression in the blood may represent the activity in the brain, which may involve the pathogenesis of psychiatric disorders, including PTSD and MDD. In support of this, dysregulation of UBE2L3 gene expression has been shown in the postmortem brain tissue sample from SZ patients which modulated mitochondrial and energy metabolism dysfunction (Huang et al., 2014).

We also found several genes that were uniquely hypermethylated in the analyses comparing PTSD + MDD patients to the control group, including hyperpolarization activated cyclic nucleotide gated potassium and sodium channel 2 (HCN2). HCN2 is a hyperpolarization-activated cation channel is mainly expressed in primary sensory neurons (Doan et al., 2004). In a previous study, HCN2 was found to have decreased expression in the cholinergic interneurons of the nucleus accumbens (NAc) of preclinical depressed model (Cheng et al., 2019). Additionally, overexpression of HCN2 was able to reverse depressive phenotypes in depressed mice (Cheng et al., 2019).

We also found another gene of interest, PAR-3 family cell polarity regulator (PAR3), to be uniquely hypermethylated in the analyses comparing PTSD and MDD patients to the control group. PAR3 is a member of the PARD family of genes, which are involved with cell division and polarized cell growth. Several studies have found that the PAR3 protein is correlated with AD. One study in particular found that in the absence of the PAR3 protein, there was a significant increase in the number of interactions between amyloid precursor protein (APP) and β-site APP cleaving enzyme 1 (BACE1) in hippocampal neurons (Sun et al., 2019). The APP/BACE1 convergence has been found to be involved with plaque development in AD (Sun et al., 2019). There have been several studies that have found a significant linkage between AD, MDD, and PTSD. One study found that veterans who had PTSD were more than twice as likely to develop incident dementia compared with those without PTSD as they aged (Yaffe et al., 2010). In another study, the development and onset of AD was found to be heavily influenced by MDD, especially when the first depressive symptoms occurred 25 years prior to AD onset, suggesting that depressive symptoms are very likely a risk factor for the development of AD later in life (Green et al., 2003). The results of our study, in addition to the literature, suggest that PAR3 could be a strong candidate for a biomarker related to the onset of PTSD and MDD.

We found several genes that are uniquely de-methylated cytosine sites in the MDD group that can be used for disease prediction. Among the novel genes identified here, discs large associated proteins 2 (DLGAP2) and PTPRN2 have been associated with psychiatric conditions (Chertkow-Deutsher et al., 2010). DLGAP2 is a member of the family of discs large associated proteins (DLGAP1-DLGAP5) located in the postsynaptic density of neurons, which plays an important role in the neuronal cell signaling and synapse organization (Rasmussen et al., 2017). Hypermethylated cytosine sites of DLGAP2 gene has been associated with neuropsychiatric disorder including autism and SZ disorder (Nardone et al., 2014; Soler et al., 2018). Upregulation of DLGAP2 were associated with synaptic malfunction and lead to maladaptive behavior in a preclinical study showing that when intron 4 in DLGAP2 was hypomethylated, rats developed a stronger ability to cope with stressful situations (Chertkow-Deutsher et al., 2010). The results of this study, as well as our findings, indicate that changes in methylation patterns in DLGAP2 are likely involved in the ability to adapt to environmental stress, making it a likely biomarker candidate for PTSD. Additionally, PTPRN2 has been correlated with several psychiatric conditions. Specifically, one study found that both PTPRN2 and DLGAP2 contained a large number of variable number of tandem repeats that were expressed in the brain, some of which ultimately correlated with attention-deficit hyperactivity disorder, SZ, bipolar disorder, and depression in relation to cocaine addiction (Linthorst et al., 2020). These results of the previous study, in addition to our current findings, imply that increased expression of both of these genes could result in increased risk of psychiatric conditions. Therefore, both DLGAP2 and PTPRN2 are viable biomarker candidates for PTSD and MDD.

While we did provide novel insights on potential biomarkers of PTSD, its application should be limited due to the small sample size. By increasing the sample size and including multiple additional time points that occurred after the initial trauma, we would be able to analyze how these genes were impacted by the event more thoroughly, which would have given us a better idea of how these serial epigenetic changes impact the genome over time in patients who express symptoms of PTSD. In this work, we utilized bisulfite sequencing and DNA methylation analysis to identify over 30 genes that could serve as potential biomarkers for the onset and prevention of PTSD and MDD. Based on our findings, we believe these genes should be studied further to develop a clearer understanding of how they affect the onset of PTSD, and how they may be used in therapeutics and diagnostics to reduce the risks for PTSD or MDD development.

## Data Availability Statement

The datasets presented in this study can be found in online repositories. The names of the repository/repositories and accession number(s) can be found below: https://www.ncbi.nlm.nih.gov/, GSE178128.

## Ethics Statement

The study was conducted according to the guidelines of the Declaration of Helsinki, and ap-proved by the Institutional Review Board of Ethics Committee at Oregon Health and Sciences University (protocol code 00812, approved 06/2014). The patients/participants provided their written informed consent to participate in this study.

## Author Contributions

CM, H-SK, and JG: conceptualization and methodology. SY: formal analysis and data curation. CM, RV, SY, H-SK, and CL: investigation. CM: writing—original draft preparation. CM, RV, MS, H-SK, SY, CL, and JG: writing—review and editing. H-SK and JG: supervision. JG: funding acquisition. All authors have read and agreed to the published version of the manuscript.

## Conflict of Interest

SY was employed by Yotta Biomed LLC. The remaining authors declare that the research was conducted in the absence of any commercial or financial relationships that could be construed as a potential conflict of interest.

## Publisher’s Note

All claims expressed in this article are solely those of the authors and do not necessarily represent those of their affiliated organizations, or those of the publisher, the editors and the reviewers. Any product that may be evaluated in this article, or claim that may be made by its manufacturer, is not guaranteed or endorsed by the publisher.
