# Supplementary figures and images for: Identification of DNA Methylation Changes That Predict Onset of Post-traumatic Stress Disorder and Depression Following Physical Trauma
Source: Front Neurosci. 2021 Sep 24;15:738347. doi: 10.3389/fnins.2021.738347 (PMC8498101; doi:10.3389/fnins.2021.738347)

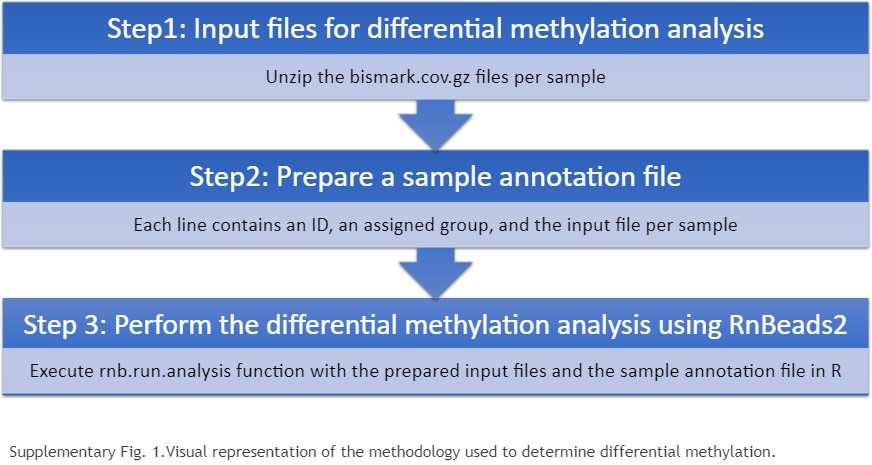

Supplement: Supplementary file 1 [file Image_1.jpg]
