# Supplementary material for: Identification of DNA Methylation Changes That Predict Onset of Post-traumatic Stress Disorder and Depression Following Physical Trauma
Source: Front Neurosci. 2021 Sep 24;15:738347. doi: 10.3389/fnins.2021.738347 (PMC8498101; doi:10.3389/fnins.2021.738347)
Supplement: Supplementary file 2 [file Table_1.docx]

**Supplementary Table 1. Gene list of hyper- and hypo-methylated cytosine sites for PTSD and MDD vs. control**

| Genes with hypermethylated cytosine sites | Genes with hypomethylated cytosine sites |
| --- | --- |
| ABR, ACAD10, ACVR1C, ADARB2, ADGRV1, ADK, ALPP, ANAPC10, ANKRD27, ANXA4, APC, APLF, ARF3, ASAH1, ASB16-AS1, ASIC2, ASIP, ATP2A3, ATP2C1, ATP4B, ATXN10, BAHCC1, BBX, BPGM, C18orf25, C4orf50, CA10, CACNA1I, CACNB2, CALCRL, CARD8, CASC21, CBS, CCDC12, CCDC144B, CCND3, CCNYL3, CDC14B, CDC42BPA, CENPP, CFAP251, CFAP46, CGB3, CHRFAM7A, CLIC4, CNTN3, CNTN5, CNTNAP3, CNTNAP3B, COBL, COL13A1, COL14A1, COL17A1, COL23A1, COMMD4P2, COPS9, CRAMP1, CYREN, DACT3, DEFB105A, DHRS9, DIP2C, DNM1P41, EIF3C, EIF3C, ENPP7P13, EP400, ERI1, ERN1, FAM155A, FAM230D, FAM230E, FAM230H, FAM230J, FAM66E, FAM85B, FAR2P1, FBRSL1, FBXO46, FCGBP, FGF17, FILIP1, FNDC3A, FOXD1, FSTL5, GABBR2, GALNT18, GATA4, GATD3A, GCC2, GINS3, GLDC, GOLGA6L22, GOLGA6L3, GOLGA6L4, GOLGA8CP, GOLGA8EP, GOLGA8T, GPHN, GRIP1, GUSBP16, GUSBP16, HCG17, HCG22, HERC2P3, HOMER2, HPS4, HSD17B7P2, HYDIN, HYDIN2, ICAM5, IGSF9, IL12B, INTS3, IPO8, IQGAP3, KCNS3, LAMA5, LDLRAD3, LHFPL3, LIMK1, LIMS3-LOC440895, LINC00313, LINC00319, LINC00535, LINC00673, LINC00965, LINC01001, LINC02028, LINC02408, LOC100130207, LOC100506639, LOC100506990, LOC100996643, LOC101927502, LOC101928195, LOC101929583, LOC102723655, LOC102724219, LOC102724788, LOC105379143, LOC110091777, LOC283194, LOC646214, LPP, LRRN1, LSP1P5, LZTS1, MAGI1, MAGI2, MANEAL, MCTP1, METTL21A, MIR548XHG, MORC1, MROH6, MRTFA, MSRA, MUC4, MVB12B, MYO16, MYO1B, MYOF, NCF1C, NCOA3, NCOA6, NEK4, NELL1, NGEF, NKD2, NPIPA2, NPIPA8, NPIPB3, NPM1, NR2F2, NTM, NUTM2A, NUTM2A-AS1, OPCML, OSBPL5, PCDHGA1, PDE10A, PDE4DIP, PDE4DIPP1, PDE4DIPP2, PDE6B, PDE8A, PDGFRA, PDPK1, PGA4, PI4KAP2, PIKFYVE, PKD1P1, PKD1P5-LOC105376752, PLA2G10, PLA2G4F, PLCG2, PLCH1, PLGRKT, PLPP1, POLR2J3, POMT1, POTEF, PRAMEF25, PRKACA, PTCSC2, PTPRN2, PYY, QSER1, RABEP1, RAD52, RALYL, RAPGEF6, RASA4B, RBFOX1, RBL1, RCOR1, REC114, REXO1L2P, RGPD5, RGR, RGS7, RIMBP2, RIN3, RNF216P1, RORB, RP1L1, RPS6KA2, RRAS2, RUFY2, S1PR5, SCFD2, SERF1A, SETBP1, SHISA6, SIMC1, SLC22A23, SLC37A3, SLC41A2, SLC45A1, SLC66A2, SLC6A4, SLC6A5, SMG1P2, SMG1P3, SMIM14, SOCS1, SPDYE10P, SPOCK1, SRGAP2-AS1, SRGAP2C, STON1, SUSD5, SYNE3, TAB2, TASOR2, TBC1D22A, TBL3, TBX2-AS1, TCAF2, TCF3, TERF2, TET1, TET3, TFDP1, THSD7B, TMCO3, TMEM132D, TNFRSF10B, TOMM7, TP73, TRAPPC10, TRAPPC13, TRAPPC9, TRPM3, TTC17, U2AF2, UBE2L3, UQCC1, USP17L11, USP17L20, USP17L24, USP28, VAV2, VPS9D1, WT1, ZC3HAV1, ZDHHC11B, ZFAT, ZMYM5, ZNF277, ZNF664-RFLNA, ZNF786, ZP3 | ACTN1, ADAM23, ADGRE1, AGBL3, AIG1, AK8, AMIGO2, ANKRD44, ANO1, ARHGAP15, ASTN2, BARD1, BCL7B, BMPER, BPIFC, C13orf42, C5AR2, CANT1, CC2D2A, CCDC26, CDH22, CHD6, CLCF1, CLDN14, CLSTN2, COL5A1, CR1L, CSGALNACT1, CSMD1, CYP2B7P, CYTH3, DBH, DDX47, DIP2A, DMXL1, DNAH6, DNHD1, DOCK4, EBF1, EBPL, EFR3B, EGFR, EPCAM-DT, ERC2, ERICH6, EVC, EYS, FAM131B, FAM160A1, FCRLB, FGF6, FGF7P3, FN3K, FNDC3B, FSHR, GABRR2, GALNT9, GAS7, GCSH, GNB1, GPC5, GRIK1, HECW1, ICAM1, INTS4P2, ITGA4, ITSN1, JAZF1-AS1, KAZN, KCNIP4, KHDRBS2, KIAA1755, LDLRAD4, LHFPL2, LINC00348, LINC00407, LINC00437, LINC00551, LINC00882, LINC00927, LINC00971, LINC01189, LINC01310, LINC02542, LOC100288637, LOC100507412, LOC101929950, LOC102724159, LOC105377975, LOC107985856, MACROH2A2, MAD2L1BP, MAP2K5, MAST3, MED12L, MFF, MIR2052HG, MIR4300HG, MIR99AHG, MS4A10, NCAM2, NEBL, NEDD4L, NLK, NRCAM, OR7E47P, OVOS2, PALM2AKAP2, PKNOX2, PLA2G6, PLD5, PLEKHA7, PPP5D1, PRIMA1, PRKAG2, PRKCE, PRKG1, PSMG2, PTCHD4, PTPRT, RAG1, RASGRP3, RBM20, RBPMS, RIMBP3B, RIMS1, RNGTT, SCEL, SDK1, SERPINA9, SETD1B, SLC14A2, SLC7A8, SLIT2, SMOC1, SORCS3, SOX2-OT, SYN3, SYT7, TBC1D3C, TCERG1L, TENM2, TENM3, TEX101, THSD7A, TLE6, TMCO1, TMTC1, TNIK, TPH2, TPRXL, TRIM49B, TSBP1-AS1, XPC, ZMYM1, ZNF423, ZNF492, ZNF532, ZNF658B, ZNF707 |
